# Psychometric properties of the exercise self-efficacy scale in patients with chronic obstructive pulmonary disease

**DOI:** 10.1371/journal.pone.0327731

**Published:** 2025-07-11

**Authors:** Cristina Sacristán-Galisteo, Tamara del Corral, Ibai López-de-Uralde-Villanueva, M Carmen Gómez, Patricia Martín-Casas

**Affiliations:** 1 Las Fronteras Healthcare Center, Primary Care Assistance Management, Madrid, Spain; 2 Doctoral Program in Healthcare, Faculty of Nursing, Physiotherapy and Podiatry, Complutense University of Madrid, Madrid, Spain; 3 Department of Radiology, Rehabilitation and Physiotherapy, Faculty of Nursing, Physiotherapy and Podiatry, Complutense University of Madrid, Instituto de Investigación Sanitaria del Hospital Clínico San Carlos (IdISSC), Madrid, Spain; 4 Department of Nursing and Physiotherapy, Alcalá University, Madrid, Spain; Niigata University of Health and Welfare: Niigata Iryo Fukushi Daigaku, JAPAN

## Abstract

**Objectives:**

This study explores the psychometric properties of the Exercise Self-Efficacy (ESE) scale among individuals with Chronic Obstructive Pulmonary Disease (COPD). The secondary objective was to evaluate the relationship between self-efficacy, functional exercise capacity, health-related quality of life and psychological state in the same population.

**Methods:**

This cross-sectional study was carried out in primary care centers in Spain. The sample was comprised of 156 people with COPD in whom anthropometric and clinical information was recorded. The ESE scale and other measures on functional exercise capacity, psychological state and health-related quality of life were administered. Psychometric properties were assessed through construct validity (EFA, exploratory factor analysis; CFA, confirmatory factor analysis), internal consistency, floor/ceiling effects and convergent validity. In this way, a quantitative, predictive and correlational research was carried out.

**Results:**

The EFA ruled out the structure of a single factor (Barletts’s test p < 0.001, Kaiser-Meyer-Olkin 0.9). ACF suggested that the most appropriate adjustment model was the two-factor solution (comparative fit index = 0.967, Tucker–Lewis index = 0.961, root mean square approximation = 0.075, standardized root mean square residual = 0.058). Thus, ESE scale is best interpreting using 15 items distributed in 2 factors (“physical and occupational/environmental barriers” and “psychosocial barriers”) than explained to 56.7% of the variance. With regard to reliability, Cronbach’s alpha was = 0.92 and no floor or ceiling effects were observed. The scale showed a moderate/strong correlation with functional exercise capacity (r = 0.41), health-related quality of life (r= − 0.53) and psychological status (r= − 0.63).

**Conclusions:**

The psychometric testing of the ESE scale provided support for the reliability and validity of the instrument in individuals with COPD. The ESE scale score shows a moderate positive correlation with functional exercise capacity and a negative correlation with health-related quality of life. In addition to a strong correlation with psychological status. In future research, the predictive power of the ESE scale in adherence to non-pharmacological treatment could be studied.

## Introduction

Chronic Obstructive Pulmonary Disease (COPD) is a heterogeneous lung condition characterized by chronic respiratory due to abnormalities of the airways and/or alveoli that cause persistent and progressive, airflow obstruction [1]. Due to the chronic nature of the disease, self-care and long-term adherence to treatment seems to be one of the keys to the correct management of the disease and the prevention/maintenance of functional deterioration and complications linked to it [2].

Among the non-pharmacological treatments, the practice of regular physical exercise stands out, which has been shown to reduce the number of exacerbations, decreased dyspnoea, fatigue and depression levels as well as improve quality of life [3]. Additionally, low levels of physical activity and a sedentary lifestyle are linked to an increased risk of mortality, hospitalization, and further disease progression [3,4].

Despite the multiple benefits of regular physical exercise in people with COPD, there is a natural progressive decrease in the level of physical activity in the long term, which is why lack of adherence constitutes one of the main challenges in the management of this sickness [5].

A determining factor in maintaining good physical exercise behavior is the level of perceived self-efficacy, which is defined as the confidence that people have in their ability to perform physical exercise in the face of different barriers [6]. Therefore, self-efficacy influences the way people act, the amount of effort they dedicate to carrying out an activity and the maintenance of a behavior over time [7].

People with COPD have lower levels of self-efficacy than the general population, as occurs in other chronic diseases such [8,9]. This is an important characteristic, as self-efficacy is positively related to the degree of adherence to pulmonary rehabilitation program, level of physical activity and functional exercise capacity [10,11]. Greater self-efficacy is correlated with a higher level of physical activity, greater distance in exercise tolerance tests, as well as has a moderate association with health-related quality of life [11,12]. In addition, high levels of depression are related to lower self-efficacy for exercise [13].

In 2005, Bandura developed the Exercise Self-Efficacy (ESE) scale which allows measuring the degree of confidence that the patient has in performing their exercise routine regularly by evaluating different situations that may make compliance difficult [6]. The original scale was composed of 18 items with a unique factor structure and high internal consistency. Subsequently, Cornick evaluated the factor structure in the general population and determined that the bifactor model provided the most precise information [14]. This scale has been translated into different languages and its psychometric properties have been studied in different chronic pathologies such as diabetes, heart or kidney disease. Thus, ESE scale has proven to be a valid and reliable tool in all of them, however the factor structure varies from 1 to 3 factors according to the different studies [15–18]. Currently, the validation of the ESE scale has not been performed in individuals with COPD. As the validity of scale is not a property of tool itself, but of the interpretation of the assessment instrument with a specific population, the validation of the ESE scale in individuals with COPD was need [19]. This would lead to objectively determining their level of self-efficacy for exercise and evaluating the barriers/limitations that could be behind the inactivity levels in individuals with COPD. Identifying the level of self-efficacy is key to understanding and predicting behavior, which would allow treatment programs to be adapted to individual’s needs, avoiding abandonment of prescribed exercise patterns [20]. Therefore, we considered the development and evaluation of the psychometric properties of the ESE scale in individuals with COPD to be very relevant.

For these reasons, the main aim of the current study was to explore the psychometric properties of the ESE scale in individuals with COPD. The secondary objective was to evaluate the relationship between self-efficacy, functional exercise capacity, health-related quality of life, as well as between psychological status.

## Methods

### Design, settings and participants

This validation study utilized a descriptive, cross-sectional design that was carried out according to the criteria established by the COSMIN guidelines (COnsensus-based Standards for the selection of health Measurement INstruments). This study was approved by the local ethics committee of Clínico San Carlos Hospital (Madrid) (22/126-EC). Participants received written information and the nature of the study was verbally explained to them. Besides, all participants expressed their free and voluntary agreement to participate in the study by signing the written informed consent.

The study population was recruited from September 4, 2022 to December 18, 2023, in different primary care centers using a non-probabilistic convenience sampling method.

The inclusion criteria for participation were: 1) confirmed diagnosis of COPD; 2) age ≥ 18 years; 3) clinical stability. The exclusion criteria were: 1) difficulty communicating in Spanish; 2) diagnosed of psychiatric or cognitive disorders that make it difficult to understand orders; 3) inability to walk independently.

### Study procedures

Evaluation required the attendance of the participants at the primary care center in two visits. At the first visit, individuals interested in participating were informed about the purpose of the study and a researcher checked whether they met eligibility criteria for the study. During the second visit, clinical characteristics were collected, as well as the functional exercise capacity, self-efficacy levels, health-related quality of life status, and anxiety and depression levels were evaluated. All the measurements were collected by face-to-face interview that during the hour-long session.

### Outcomes

#### 1. Anthropometric and clinical characteristics.

Anthropometric characteristics and clinical characteristics, the Body Mass Index (BMI), smoking habits (pack/year index), number of comorbidities (Charlson Comorbidity index), lung function (forced spirometry test), COPD severity (Global Initiative for Chronic Obstructive Lung Disease; GOLD), COPD prognosis (BMI, airflow Obstruction, Dyspnea, Exercise capacity; BODE index), dyspnoea (modified medical research council; mMRC) and lower limb fatigue (modified Borg scale), as well as medication were recorded during the interview.

#### 2. Self-efficacy.

Self-efficacy was analyzed using the ESE scale which evaluates the subject´s degree of confidence in performing exercise 3 or more times per week, taking into account different barriers and obstacles. It is composed of 18 items that are valued from 0 to 100 points (0 “not being able to do it at all” and 100 “very sure I can do it”). Total score is obtained by adding all the items and a higher score indicates higher self-efficacy for exercise [6].

#### 3. Functional exercise capacity.

Functional exercise capacity was evaluated by the Six-Minute Walk distance (6MWD).

#### 4. Anxiety and depression levels.

Distress was measured by the Hospital Anxiety and Depression Scale (HADS). The cut-off point to consider a case was ≥ 12 [21].

#### 5. Health-related quality of life.

To assess the participants’ quality of life, we employed the Spanish version of the COPD Assessment Test (CAT) [22].

### Sample size

Initially, a minimum sample size of 144 individuals with COPD was established to be able to perform an adequate exploratory factor analysis (EFA) and confirmatory factor analysis (CFA) [23–27], since some authors consider it necessary to have at least 8 cases per observed variable to reach an adequate sample size [28]. In addition, several authors have established that in scales with more than 3 items per factor, a sample of at least 150 individuals would be sufficient to be able to generate reliable results about the theoretical structure of the scale [23,29,30]. Thus, it was finally decided to reach a sample size of at least 150 individuals to satisfy both criteria.

### Data analysis

SPSS software and the psych and lavaan packages in R were used to conduct all the statistical analyses. Statistical significance was set at 5% (*p* < .05).

### Construct validity: Exploratory and confirmatory factor analysis

Construct validity was assessed through a two-step process. Firstly, an EFA was performed to identify the optimal factor structure of the ESE scale. Thus, to determine whether Pearson’s correlation matrix was factorizable, the Barlett’s test and the Kaiser-Meyer-Olkin (KMO) test were examined [31]. The Kaiser’s eigenvalue criterion (eigenvalue ≥1) and the parallel analysis method were used to establish the optimal number of factors [32]. A principal axis factoring method with oblimin rotation was employed as the factor extraction method, since this rotation allows the factors to be correlated. A factor loading of >0.4 was considered necessary for the item inclusion. Secondly, to confirm the theoretical factor structure, a confirmatory factor analysis (CFA) was performed using the robust unweighted least squares (ULSMV) estimation method. For model fit evaluation, comparative fit index (CFI) ≥.90, Tucker-Lewis index (TLI) ≥ 0.90, root mean square error of approximation (RMSEA) ≤.08, and standardized root mean square residual (SRMR) ≤.10 were used as indicators of acceptable fit [33,34]. Both exploratory and confirmatory factor analyses were used to strengthen the internal validity of the study and to increase confidence in the underlying structure of the evaluated construct.

### Floor/ceiling effects and reliability

The presence of floor and/or ceiling effect was assumed if at least 15% of participants achieved the minimum and/or maximum score [35]. The reliability of the ESE scale in individuals with COPD was assessed by internal consistency, with a value of > 0.7 being considered acceptable [36].

### Convergent validity

Convergent validity was assessed by Pearson correlations between the ESE scale and the other variables.

## Results

### Sample description

Baseline anthropometric and clinical characteristics of the sample are presented in **Table 1**.

**Table 1:** Baseline anthropometric, smoking habits and clinical characteristics of the sample (n = 156).

	Mean ± SD	N (%)
**Anthropometric characteristics**		
Age (years)	70.45 ± 7.89	
Female		46 (29%)
BMI (kg/m^2^)	28.47 ± 5.28	
**Smoking habits**		
Current smoker		60 (38%)
Smoking history (pack/years index)	49.55 ± 31.06	
**Clinical characteristics**		
FEV1 (% predicted)	63.13 ± 15.45	
FEV1/FVC	57.96 ± 9.01	
COPD severity (GOLD)	2.08 ± 0.56	
Mild		16 (10%)
Moderate		113 (72%)
Severe		25 (16%)
Very severe		2 (1%)
COPD prognosis (BODE index)	1.36 ± 1.47	
Comorbidities (Charlson Comorbidity Index)	1.95 ± 1.05	
Functional exercise capacity (6MWD)	443.07 ± 105.02	
Dyspnoea (mMRC grade 0–4)	1.1 ± 0.78	
Grade 0		36 (23%)
Grade 1		73 (47%)
Grade 2		43 (28%)
Grade 3		4 (3%)
Grade 4		0 (0%)
Lower limb fatigue (modified Borg scale)	2.13 ± 3.02	
Health-related quality of life (CAT)	11.31 ± 7.7	
Psychological status (HADS)		
Anxiety (HADS-Anxiety)	4.68 ± 4.24	
Depression (HADS-Depression)	4.16 ± 4.5	
Distress (HADS-Total)	8.84 ± 7.78	
**Medications**		
Inhaled corticosteroids		89 (57%)
Long-acting bronchodilators (LAMA or LAMA)		152 (97%)

**Abbreviatures:** 6MWD, Six-min walk distance; BODE, Body mass index, degree of airflow obstruction, dyspnoea, exercise capacity; BMI, Body mass index; CAT, COPD Assessment Test; COPD, Chronic obstructive pulmonary disease; FEV_1_, Forced expiratory volume at the first second; FVC, Forced vital capacity; GOLD, Global initiative for chronic obstructive lung disease; HADS, Hospital anxiety and depression scale; LAMA, Long acting muscarinic antagonist; LABA, Long acting beta agonist; mMRC, Modified medical research council.

### Construct validity

#### Exploratory factor analysis.

Preliminary analyses of the initial set of 18 items showed that all items contributed to the scale (Cronbach’s alpha = 0.93; adjusted item-total correlations = 0.497–0.787, p < .05). Bartlett’s test of sphericity rejected the null hypothesis of identity matrix (χ^2^ (153) = 1895.20, *p* < .001), as well as an adequate value on the KMO (0.90), suggesting adequate data to perform an EFA. Both Kaiser’s eigenvalue criterion and the parallel analysis method suggested retaining 2 factors, which account for 54.6% of the variance explained. However, the preliminary AFE showed the presence of 4 problematic items. Items 16 and 18 were not very reliable observed indicators, as they presented a very low communality (≤ 0.3) and the factor loadings of these items showed great similarity for both factors which could indicate that they do not contribute significantly to either factor. On the other hand, items 13 and 17 presented loadings in the matrix without rotation in both a first factor and a second factor generated only by these two items. In a more detailed analysis, it was observed that both items presented a very high correlation (r = 0.87), which suggests that they are redundant items and could explain why only these two items generated a second factor in the matrix without rotation. For these reasons, it was decided to eliminate items 16, 17 (eliminated in place of 13 because it had a lower measure of sampling adequacy [MSA]) and 18. Thus, the final model with 15 items was distributed in 2 factors (accounting for 56.7% of the variance). Factor 1, labelled “physical and occupational/environmental barriers”, consisted of items 1, 2, 3, 4, 8, 9, 11 and 14. Factor 2, labelled “psychosocial barriers”, consisted of items 5, 6, 7, 10, 12, 13 and 15. The factor loading of each item in the EFA is shown in **Table 2**.

**Table 2:** Final exploratory factor analysis solution.

Item	Physical and occupational/ environmental barriers	Psychosocial barriers
**1. When I am feeling tired.** ***Cuando me siento cansado.***	.724*	–
**2. When I am feeling under pressure from work.** ***Cuando me siento presionado por el trabajo.***	.757*	–
**3. During bad weather.** ***Durante el mal tiempo.***	.689*	–
**4. After recovering from an injury that caused me to stop exercising.** ***Después de recuperarme de una lesión que hizo que dejara de hacer ejercicio.***	.702*	–
**5. During or after experiencing personal problems.** ***Durante o después de pasar por problemas personales.***	–	.715*
**6. When I am feeling depressed.** ***Cuando me siento deprimido.***	–	.772*
**7. When I am feeling anxious.** ***Cuando me siento ansioso.***	–	.743*
**8. After recovering from an illness that caused me to stop exercising.** ***Después de recuperarme de una enfermedad que hizo que dejara de hacer ejercicio.***	.795*	–
**9. When I feel physical discomfort when I exercise.** ***Cuando siento incomodidad física cuando hago ejercicio.***	.726*	–
**10. After a vacation.** ***Después de las vacaciones.***	–	.790*
**11. When I have too much work to do at home.** ***Cuando tengo demasiado trabajo para hacer en casa.***	.839*	–
**12. When visitors are present.** ***Cuando tengo visitas.***	–	.677*
**13. When there are other interesting things to do.** ***Cuando hay otras cosas interesantes que hacer.***	–	.854*
**14. If I don´t reach my exercise goals.** ***Si no alcanzo mis metas en el ejercicio.***	.724*	–
**15. Without support from my family or friends.** ***Sin el apoyo de mi familia o amigos.***	–	.717*
**16. During a vacation.** ***Durante las vacaciones.***	–	–
**17.When I have other time commitments.** ***Cuando tengo otros compromisos.***	–	–
**18. After experiencing family problems.** ***Después de pasar por problemas familiares.***	–	–

*Note:* * = *p *< .05.

#### Confirmatory factor analysis.

**Fig 1:**
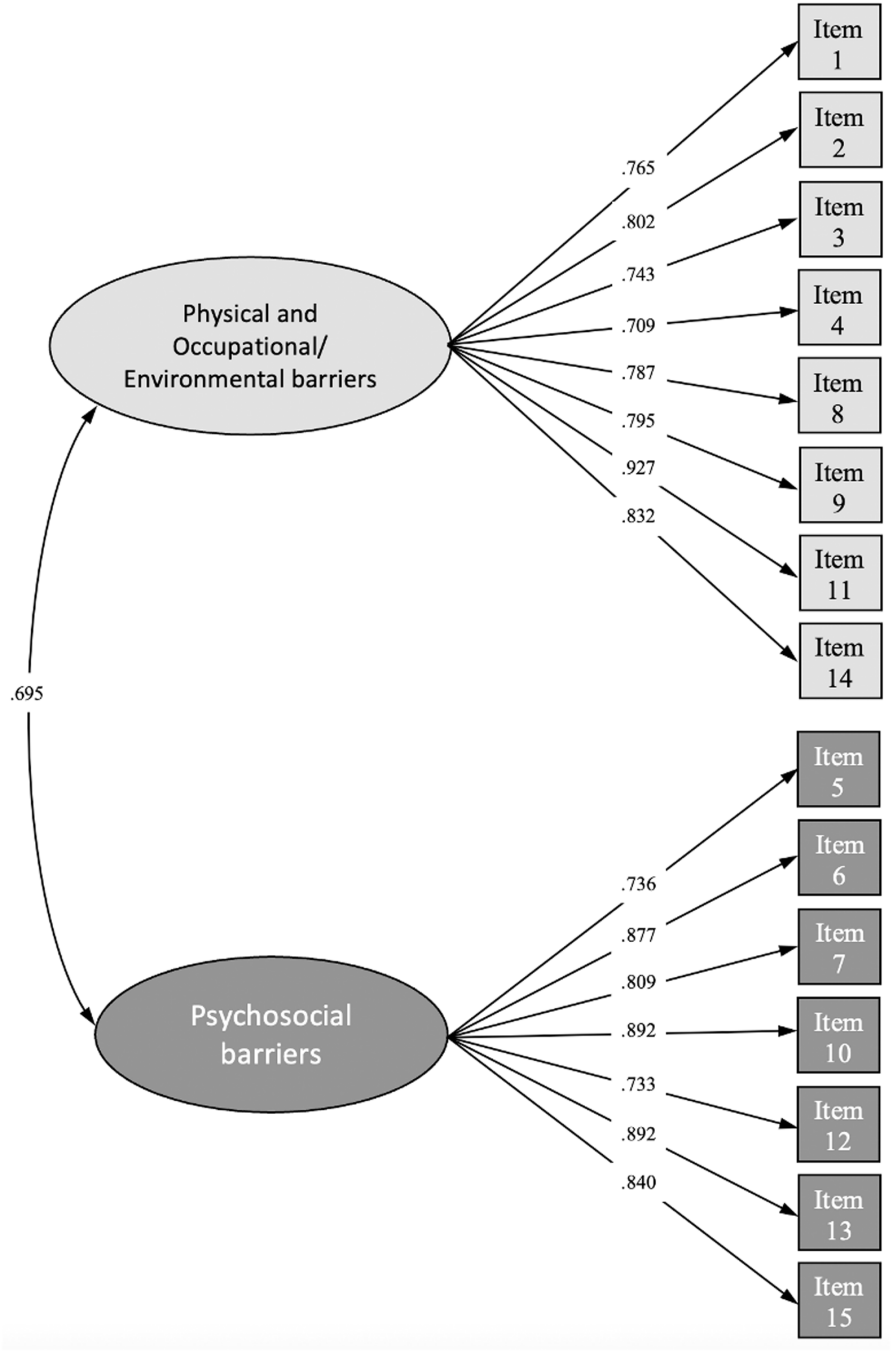
Structural equation modeling for the final 15-item version of the Bandura’s exercise self-efficacy scale.

According to CFA, the goodness-of-fit indices showed that 2-factor model fit well with the data: CFI = 0.967, TLI = 0.961; SRMR = 0.058; RMSEA = 0.075, 90% CI = 0.058 to 0.093. **Fig 1** shows the standardized factor loadings of this final higher-order 15-item 2-factors solution. Finally, the two factors were strongly correlated (r = 0.695; *p* < .001).

### Floor and ceiling effects and reliability

The final 15-item version of the ESE scale for individuals with COPD consisted of 15 items formulated in a direct/positive manner, which were distributed across 2 factors/subconstructs. Each item is scored on a scale of 0–100 points ranging in 10–point intervals, so that the total score of the final version of the ESE scale for people with COPD ranges from 0 to 1500 points. No floor or ceiling effect was detected in the final version of the ESE scale, as only 5 participants obtained the lowest or highest possible score (floor = 1%; ceiling = 3%).

The final 15-item version of the ESE scale was excellent (Cronbach’s alpha = 0.92; 95% CI = 0.91 to 0.94), and its 2 factors/subconstructs also showed adequate internal consistency (“physical and occupational/environmental barriers”, 0.91 [0.88–0.93]; “psychosocial barriers”, 0.90 [0.87–0.92]).

### Convergent validity

The correlations of the 15-item version of the ESE scale with all the other variables are shown in **Table 3**.

**Table 3:** Pearson correlations of exercise self-efficacy (ESE) scale with clinical characteristics.

Outcome	ESE scale 15-item		
	TOTAL	Psychosocial barriers	Physical and occupational/ environmental barriers
**COPD severity**			
GOLD	–0.23^**^	–0.23^**^	–0.19^*^
**COPD prognosis**			
BODE index	–0.42^**^	–0.38^**^	−0.37^**^
**Functional exercise capacity**			
6MWD (m)	0.41^**^	0.38^**^	0.36^**^
**Symptomatology**			
Dyspnoea (mMRC)	–0.44^**^	–0.42^**^	–0.36^**^
Lower limb fatigue (modified Borg scale)	–0.29^**^	–0.31^**^	–0.22^**^
**Health-related quality of life**			
COPD assessment test	–0.53^**^	–0.47^**^	–0.47^**^
**Psychological status**			
Anxiety (HADS-Anxiety)	–0.51^**^	–0.49^**^	–0.43^**^
Depression (HADS-Depression)	–0.61^**^	–0.56^**^	–0.53^**^
Distress (HADS-Total)	–0.63^**^	–0.59^**^	–0.54^**^

**Abbreviatures:** 6MWD, Six-min walk distance; BODE, Body mass index, degree of airflow obstruction, dyspnoea, and exercise tolerance; COPD, Chronic obstructive pulmonary disease; GOLD, Global initiative for chronic obstructive lung disease; HADS, Hospital anxiety and depression scale; mMRC, modified medical research council.

* *p* value < .05.

** *p* value < .01.

The total score of the ESE scale presented a moderate/strong correlation with all the variables evaluated (r = |0.41–0.63|; *p* < 0.01) except with GOLD and lower limb fatigue where a low correlation was found (r < |0.30|; *p* < 0.01). All correlations were inverse except the functional exercise capacity. Thus, people with COPD with greater perceived self-efficacy presented less severity of the pathology, dyspnoea, lower limb fatigue, impact on their health-related quality of life and distress. The only direct correlation was found with the 6MWD (r = 0.41; *p* < 0.01), where participants with a higher degree of perceived self-efficacy walked a greater distance. Finally, it should be noted that both the “physical and occupational/environmental barriers” subscale and the “psychosocial barriers” subscale showed correlations of similar magnitude with all the variables evaluated, these magnitudes being slightly higher the correlations observed with the “psychosocial barriers” subscale.

## Discussion

The ESE scale has emerged as a valid and reliable tool to measure the level of exercise self-efficacy in individuals with COPD, according to the evaluation of its psychometric properties. The brevity of the ESE scale, the ease of administration, the simplicity of the language, and scoring are likely to fit with the needs of clinicians for brief but reliable screeners, that can be easily and flexibly administered.

The final version of the scale was composed of 15 items divided into two factors: “physical and occupational/environmental barriers” and “psychosocial barriers”. This factorial structure was similar to that detected in Spanish individuals with metabolic syndrome and in the general population [14,17]. In the factor analysis, the scale showed a wonderful level of inter-correlation of its items, similar to that found in cardiac rehabilitation patients [18]. However, the preliminary AFE showed the presence of 4 problematic items. Items 13 (“When there are other interesting things to do”) and 17 (“When I have other time commitments”) presented loadings in the matrix without rotation in both a first factor and second factor. This was due to the fact that both items had the high level of correlation which suggests that they are redundant and it is possible that patients interpret both concepts as similar. In this way, it was decided to eliminate item 13 because it had a lower measure of sampling adequacy. On the other hand, items 16 (“During a vacation”) and 18 (“After experiencing family problems”) were removed due to exhibiting low communality values (<0.3), indicating that the proportion of variance explained by the two factors was limited. Furthermore, the factor loadings of these items were very similar across both factors. This could suggest, a priori, the generation of a third factor to include these two items. However, the exploratory factor analysis indicates that the most suitable fit model is with two factors. This is corroborated by the internal consistency analysis, which shows that when both items are removed, the internal consistency increases in both factors instead of decreasing. Thus, as these items are problematic, it was decided to eliminate them. Others authors have also found that interpersonal factors have proven to be some of the most difficult barriers to overcome [16]. In the present study, considering that the mean age of the sample was 70 years, and therefore the majority of this population was retired, it would be reasonable to think that being on vacation might not be a barrier to exercise in this population. Regarding the elimination of item 18, its low factor loading could be explained by the cultural context due to the family-oriented nature of Spanish society, based on family values and a strong sense of responsibility for caregiving; in similar contexts, family problems have proven to be one of the most difficult interpersonal factors for exercise to overcome, since they are situations in which the person have less direct influence and therefore less control [37–39]. The elimination of items 13 and 16 also occurred in the validation of the scale in individuals with metabolic syndrome [17,40].

The absence of floor and ceiling effect suggests that the items represent great variability in different situations that can make it difficult to comply with regular exercise. This lack of ceiling-floor effect has been corroborated in other previous validation studies [16,18,40].

As a whole, the scale showed great reliability with excellent internal consistency, which agrees with the results obtained in other chronic pathologies such as heart disease or metabolic syndrome [17,18].

Regarding convergent validity, the score on the ESE scale showed a moderate to strong correlation with health-related quality of life, functional exercise capacity, and psychological status, which corroborates the positive relationship found in previous studies in people with COPD [11–13]. Thus, a greater sense of exercise self-efficacy implies greater confidence in exercising, and it has been associated with a greater likelihood of engaging in and maintaining this behavior over time [41]. Considering that regular physical exercise in people with COPD has been shown to benefit quality of life, functional exercise capacity, and psychological state by reducing symptoms, the correlations found between these variables seem reasonable [11,42,43].The low correlation found with the severity of the obstruction (GOLD) could be due to the fact that the degree of obstruction alone has proven to be an insufficient value in the COPD population due to its weak association with the symptoms [1,44]. This correlation has important clinical implications for the management of individuals with COPD and for treatment adherence. It has been demonstrated that a higher level of exercise self-efficacy is associated with greater attendance at pulmonary rehabilitation programs, increased adherence to medical treatment, improved quality of life, psychological status, and exercise capacity [11,12,20]. Therefore, tools to monitor exercise self-efficacy may be useful for predicting positive outcomes in these constructs and guiding specific interventions to strengthen self-efficacy levels in cases where a decline is detected regardless of the severity of the obstruction.

The brief nature of the ESE scale makes it an ideal tool for time-poor clinicians such as general practitioners. Assessing levels of exercise self-efficacy in the area of primary care setting is important because people with low level of exercise self-efficacy could be identified early. This early identification may lead to the implementation of interventions to improve exercise adherence, which has been shown to be very important for people with COPD. These interventions would enhance the self-care and health promotion, which are the main pillars of primary care.

The main limitations of the study were the lack of evaluation of the reliability test-retest to measure the temporal stability and also sensitivity to change of the scale. These evaluations were not included as objectives of the study, as it was a cross-sectional study with a single measurement. On the other hand, the non-probabilistic convenience sampling procedure used introduces a selection bias, which could limit the generalization of the results. Finally, the performance of a confirmatory factor analysis on the same sample constitutes a bias. Nevertheless, owing to the removal of several items during the exploratory factor analysis, it was considered that conducting a confirmatory analysis and illustrating certain indices could provide greater value to the study. However, these limitations should be considered in future studies.

The strengths of the study are the collection of data from a large sample in the primary care setting which allows the inclusion of individuals with clinical variability. All data were collected through a face-to-face interview by the same evaluator, to increase internal validity.

It would be relevant, in future studies, to conduct a confirmatory factor analysis on a different sample, which could corroborate the theoretical factor structure found in the present study. The use of probabilistic sampling and to increase the sample size in future validation studies would allow for more diverse samples, including different severities of COPD, wider range of age and greater sociodemographic variability, ensuring that the results of the validation of the scale are applicable to a broader and more representative population. Furthermore, it would be important to assess the temporal stability of the scale through test-retest control and the sensitivity to change of the scale. Finally, in future research, it would be also interesting to evaluate the predictive power of the ESE scale on the results in pulmonary rehabilitation and the degree of adherence to long-term non-pharmacological treatment.

## Conclusions

The ESE scale is a valid and reliable tool that can reliably estimate levels of exercise self-efficacy in the individuals with COPD. The results of the study confirm that the structure of 15 items divided into 2 factors is the most appropriate in population with COPD. Furthermore, the ESE scale score has demonstrated a moderate/strong positive correlation with functional exercise capacity and negative with health-related quality of life and psychological status. In future research, it would be interesting to evaluate whether the ESE scale can predict the level of long-term adherence to treatment of people with COPD.
